# Reduced cortical folding in multi-modal vestibular regions in persistent postural perceptual dizziness

**DOI:** 10.1007/s11682-018-9900-6

**Published:** 2018-06-02

**Authors:** Salvatore Nigro, Iole Indovina, Roberta Riccelli, Giuseppe Chiarella, Claudio Petrolo, Francesco Lacquaniti, Jeffrey P. Staab, Luca Passamonti

**Affiliations:** 1 National Research Council, Institute of Bioimaging and Molecular Physiology, Catanzaro, Italy; 2 Centre of Space BioMedicine, University of Rome TorVergata, Rome, Italy; 3 Laboratory of Neuromotor Physiology, IRCCS Santa Lucia Foundation, Rome, Italy; 4 Department of Experimental and Clinical Medicine, University MagnaGraecia, Catanzaro, Italy; 5 Department of Systems Medicine, University of Rome TorVergata, Rome, Italy; 6 Departments of Psychiatry and Psychology and Otorhinolaryngology – Head and Neck Surgery, Mayo Clinic, Rochester, MN USA; 7 Department of Clinical Neurosciences, University of Cambridge, Cambridge, CB2 0SZ UK

**Keywords:** Persistent postural perceptual dizziness, Surface based morphometry, Local gyrification index, Vestibular cortex, Occipital cortex, Superior parietal cortex

## Abstract

Persistent postural perceptual dizziness (PPPD) is a common functional vestibular disorder that is triggered and sustained by a complex interaction between physiological and psychological factors affecting spatial orientation and postural control. Past functional neuroimaging research and one recent structural (i.e., voxel-based morphometry-VBM) study have identified alterations in vestibular, visuo-spatial, and limbic brain regions in patients with PPPD and anxiety-prone normal individuals. However, no-one thus far has employed surface based morphometry (SBM) to explore whether cortical morphology in patients with PPPD differs from that of healthy people. We calculated SBM measures from structural MR images in 15 patients with PPPD and compared them to those from 15 healthy controls matched for demographics, personality traits known to confer risk for PPPD as well as anxiety and depressive symptoms that are commonly comorbid with PPPD. We tested for associations between SBM measures and dizziness severity in patients with PPPD. Relative to controls, PPPD patients showed significantly decreased local gyrification index (LGI) in multi-modal vestibular regions bilaterally, specifically the posterior insular cortices, supra-marginal gyri, and posterior superior temporal gyri (*p* < 0.001). Within the PPPD group, dizziness severity positively correlated with LGI in visual areas and negatively with LGI in the right superior parietal cortex. These findings demonstrate abnormal cortical folding in vestibular cortices and correlations between dizziness severity and cortical folding in visual and somatosensory spatial association areas in PPPD patients, which provides new insights into the pathophysiological mechanisms underlying this disorder.

**Electronic supplementary material:**

The online version of this article (10.1007/s11682-018-9900-6) contains supplementary material, which is available to authorized users.

## Introduction

Persistent postural perceptual dizziness (PPPD) is a chronic functional vestibular disorder that lies at the interface between neurology, otology, and psychiatry. Clinically, it is characterized by persistent dizziness, unsteadiness, and swaying or rocking (non-spinning) vertigo (Staab et al. 2017). These symptoms may be exacerbated by upright posture, patients’ own movements, and exposure to environments containing complex and moving visual stimuli. PPPD may be triggered by neurotologic or other medical and psychological events that cause vertigo, unsteadiness, or dizziness or disrupt balance, including peripheral vestibular disorders, vestibular migraine, panic attacks, generalized anxiety disorders, mild traumatic brain injury, and hypotensive episodes (Dieterich and Staab 2017; Staab et al. 2017).

In the midst of these events, patients shift posture and gait control from a relaxed position to a high-risk strategy of greater stiffness and shorter strides similar to what normal individuals do in situations of increased postural threat such as standing or walking at heights (Brown et al. 2002; Gage et al. 2003). Patients with acute vestibular symptoms also rely more strongly on visual or somatosensory cues than vestibular inputs for postural control and spatial orientation, a process known as visual or somatosensory dependence (Cousins et al. 2014). Normally, as patients recover from conditions that cause vestibular or balance problems, they revert back to naturally relaxed postural control and return to a more cohesive combination of vestibular, visual, and somatosensory cues for determining spatial orientation. However, patients who develop persistent dizziness fail to return to low-risk postural control and maintain high levels of visual dependence, even if they otherwise recover or fully compensate for the events that precipitated their initial symptoms (Staab 2012; Staab et al. 2013; Staab and Ruckenstein 2007). These persistent functional alterations in postural control and spatial orientation are thought to be key pathophysiologic mechanisms underlying PPPD; thus, it is considered to be a chronic functional disorder (Staab 2012).

A formal definition of PPPD was recently promulgated by the World Health Organization (World Health Organization 2016) and the Bárány Society, the international neurotologic research organization (Staab et al. 2017). However, observations of similar symptoms may be found in the original description of agoraphobia from 1871 (Westphal 1871) and debates about the relative contributions of neurologic, otologic, and psychologic factors to difficulties that certain individuals had with locomotion, spatial orientation, and avoidance behaviors in the rambunctious marketplaces of nineteenth century town squares (Balaban and Jacob 2001). Contemporary predecessors of PPPD include phobic postural vertigo (PPV) (Brandt and Dieterich 1986), space motion discomfort (Jacob et al. 1989), visual vertigo (Bronstein 1995), and chronic subjective dizziness (CSD) (Staab et al. 2004). Epidemiologic studies of these conditions indicate that PPPD is the second or third most common cause of dizziness among patients referred to tertiary neurotologic clinics (Staab 2012).

Earlier studies explored potential etiopathogenic and pathophysiological mechanisms of PPPD or its predecessors, finding that distinct personality traits confer risk for PPPD or protect against its development (Chiarella et al. 2016; Indovina et al. 2015; Staab et al. 2014). Specifically, in a study from the USA, a combination of high neuroticism and low extraversion was found in a significantly larger proportion of patients with CSD than in a comparison group of patients with other vestibular disorders who had similar levels of dizziness, anxiety, and depression (67% vs. 25%) (Staab et al. 2014). High levels of neuroticism were also reported in Italian patients with CSD (Chiarella et al. 2016) and Chinese patients with PPPD (Yan et al. 2017).On the other hand, German individuals with higher ratings of resilience, optimism, and sense of coherence experienced a lower incidence of functional dizziness after acute vestibular events (Tschan et al. 2011).

Pre-existing anxiety disorders were also linked to the development of PPV and CSD after acute neurotologic illnesses and premorbid personal or family histories of anxiety disorders were associated with poorer response to selective serotonin reuptake inhibitors (Staab and Ruckenstein 2005), the mainstay of pharmacologic treatment in PPPD. Furthermore, five prospective studies showed that high anxiety and body vigilance in the setting of acute vestibular disorders predicted persistent PPPD-like dizziness far better than measures of structural vestibular deficits, including one Australian investigation that demonstrated a durable reduction in symptoms with just three cognitive behavior therapy sessions administered within eight months of the acute illness (i.e., before chronic symptoms had consolidated) (Best et al. 2009; Cousins et al. 2017; Edelman et al. 2012; Godemann et al. 2005; Heinrichs et al. 2007). Together, these studies from four continents indicate that anxiety-related personality traits and a pre-existing anxiety diathesis are risk factors for PPPD and that high anxiety and body vigilance promote its development. In contrast, there is less evidence that anxiety-related factors are necessary to sustain PPPD after it is well established. Around 40% of patients with PPPD have no active anxiety disorders (25% have no psychiatric morbidity at all) (Staab 2012) and 8–12 sessions of cognitive behavioral therapy conducted in patients with long-standing PPV offered no lasting benefit (Holmberg et al. 2006, 2007).

Neuroimaging studies are beginning to provide evidence for the brain mechanisms by which anxiety-related personality traits may influence the processing of vestibular and visual information for spatial orientation and locomotion. Two functional magnetic resonance imaging (fMRI) studies performed in healthy individuals showed that anxiety-related personality traits affect activity and functional connectivity patterns within vestibular, visual, and limbic areas of the brain (Indovina et al. 2014; Riccelli et al. 2017a). The first investigation used vestibular stimulation from a short tone burst that activates the otoliths (Indovina et al. 2014). The second one used visual motion stimulation from an immersive virtual reality rollercoaster ride (Riccelli et al. 2017a). In the first study, higher levels of neuroticism, measured by the NEO Personality Inventory Revised (NEO-PI-R) (Costa and McCrae 1997), correlated positively with activity in the brainstem, bilateral cerebellar fastigium, and left visual cortex and negatively with activity in the left supramarginal gyrus. High levels of introversion (i.e., low extraversion scores) correlated with increased activity in the amygdala. Higher levels of neuroticism were also linked to heightened connectivity between the amygdala and brainstem, amygdala and fastigium, left inferior frontal gyrus and left supra-marginal gyrus, and left inferior frontal gyrus and left visual cortex (Indovina et al. 2014). Introversion correlated negatively with connectivity between the right amygdala and inferior frontal gyrus. In the second study, neuroticism scores correlated positively with activity in the left posterior insular cortex (PIC), another component of the non-dominant vestibular cortex, and with increased functional connectivity between the left PIC and right amygdala. Thus, in response to vestibular and visual motion stimuli, anxiety-related personality traits in normal individuals were associated with greater reactivity and connectivity in key brain regions that process vestibular, visual, and threat-related information and with increased responses in visual areas (Riccelli et al. 2017a).

Two recent fMRI studies using sound-evoked vestibular stimulation and visual motion stimulation compared patients with PPPD to a group of normal individuals matched for NEO-PI-R personality traits, anxiety and depression (Indovina et al. 2015; Riccelli et al. 2017b). Relative to controls, patients with PPPD showed reduced activation in response to vestibular stimulation of the right posterior insula and adjacent superior temporal gyrus (components of the dominant vestibular cortex) as well as in the left anterior insula extending into the frontal operculum and the left inferior frontal gyrus, the left anterior cingulate cortex, and the left hippocampus. Patients with PPPD also had more negative functional connectivity between the right superior temporal gyrus and both the left anterior cingulate cortex and left hippocampus as well as between the left anterior insula/inferior frontal gyrus and right middle occipital cortex (Indovina et al. 2015). Furthermore, patients with PPPD showed alterations in brain networks that affect balance control and reweighting of space-motion inputs to favor visual cues (Riccelli et al. 2017b). The results of a recent resting state fMRI study were consistent with these findings, showing that patients with PPPD, relative to healthy controls, had decreased connectivity between the left hippocampus and the bilateral temporal, insular, central opercular, and occipital cortices (Lee et al. 2018). Similarly, a recent structural imaging investigation found that patients with PPPD, relative to healthy controls, had decreased gray matter volume as assessed via voxel-based morphometry (VBM) in the temporal cortex, cingulate cortex, precentral gyrus, hippocampus, dorsolateral prefrontal cortex, caudate nucleus, and the cerebellum (Wurthmann et al. 2017).

Together, these data strongly suggest that PPPD may be linked to functional and structural alterations in crucial vestibular, visual, and frontal regulatory regions of the brain, including those that modulate attention and response to threat-related stimuli (Balaban and Thayer 2001; Staab et al. 2013). However, comparing the results from normal individuals showing neuroticism-associated increased activity and connectivity in these brain regions to the findings from patients with PPPD showing decreased activity and connectivity, it is clear that more information is needed about how the brain structure and function relate to the development of PPPD. In addition, the use of sophisticated structural imaging techniques, particularly surface-based morphometry (SBM), may allow differentiation of the contributions played by key neuroanatomical markers (i.e., cortical thickness, surface area, and cortical folding) to alterations in the anatomy of the cortical mantle. It also may enable to disentangle among features such as the gray-matter changes recently observed in a VBM study of patients with PPPD (Wurthmann et al. 2017). This is an important issue as cortical thickness, surface area, and cortical folding are thought to have distinct developmental trajectories and cellular mechanisms (Rakic 2009; Raznahan et al. 2011). More specifically, cortical thickness (CT) depends on the horizontal layers within the cortical columns, while the surface area (SA) relates to the number of radial columns perpendicular to the pial surface (Dale et al. 1999; Panizzon et al. 2009; Rakic 2009). In contrast, the cortical folding results from the underlying microstructure of the neuronal sheets and from the local connectivity within a cortical region (Schuez and Miller 2003; White et al. 2010).

The purpose of the current study was thus to search for morphological changes across the cortical mantle in PPPD, considering the possibility that any identified abnormalities could represent previously undetected structural causes or risk factors for the disorder or secondary structural alterations induced by persistent shifts in physiological functioning. We employed well-validated SBM techniques to examine cortical surface anatomy in patients with PPPD relative to a group of healthy controls. On the basis of previous studies (Balaban and Thayer 2001; Indovina et al. 2015; Staab et al. 2013, 2014; Wurthmann et al. 2017), we hypothesized that patients with PPPD, relative to healthy people, would show alterations in CT, SA, and cortical folding in brain regions belonging to vestibular, visual, and emotional neural networks. Specifically, we predicted abnormalities in surface morphology in the vestibular cortex (PIC, parietal operculum, posterior superior temporal gyrus, and supramarginal gyrus) (Bense et al. 2001; Bottini et al. 2001; Lacquaniti et al. 2013; Lopez et al. 2012), visual cortex (Cousins et al. 2014; Indovina et al. 2015), and frontal regions that regulate anxiety-related behaviors (inferior frontal gyrus, and anterior cingulate cortex). Informed by our previous neuroimaging work (Indovina et al. 2015), we wanted to minimize the potential confounds of psychological variables on the results of the structural analyses as there is evidence that these factors may be significantly associated with variations in cortical morphology, even in healthy people with no psychiatric disorders (Riccelli et al. 2017c). Therefore, we matched our patients with PPPD to a group of healthy controls on standardized measures of personality traits, anxiety, and depression.

## Methods

### Participants

Fifteen right-handed patients who had developed PPPD after an acute vestibular syndrome (see below for further details) were enrolled in this study. All patients had fully recovered or compensated for their peripheral vestibular conditions at the time of study entry. This cohort overlaps to the one that we investigated in our previous fMRI study of CSD. Subjects were recruited for that investigation using the diagnostic criteria for CSD, but their clinical histories were verified against the definitions of PPPD posted by the World Health Organization (http://www.who.int/classifications/icd/en) and Bárány Society, specifically: (i) one or more symptoms of non-vertiginous dizziness, unsteadiness, or swaying-rocking (non-spinning) vertigo lasting 3 months or more, (ii) symptoms present most days, throughout the day (though they may wax and wane), (iii) symptoms exacerbated by upright posture, active or passive head motion, and exposure to moving or complex visual stimuli. Exclusion criteria for this study included active neuro-otologic disorders other than PPPD, chronic medical illnesses, pregnancy, medication use, smoking, and history of head injury.

A history of quiescent or fully compensated vestibular peripheral deficits at the time of study was not an exclusion criterion. This was because otologic illnesses are known to be the most common triggers of PPPD (Staab and Ruckenstein 2003, 2007), as was the case in our patient group. In particular, most of our patients with PPPD had a history of vestibular neuritis (*N* = 12), while a few of them had experienced benign paroxysmal positional vertigo (*N* = 2) or both (N = 1). These disturbances were localized on the right side in seven patients, left side in seven patients, or bilaterally in one patient. Patients with PPPD who had vestibular neuritis underwent caloric testing in the acute stage of their peripheral vestibular disease and 6 months later to evaluate the adequacy of their recovery. The percentage of reduced vestibular response on the electronystagmogram was calculated using the Jongkees’ formula (Furman and Jacob 1993), which revealed mild to moderate unilateral canal paresis (relative vestibular reduction in the nystagmus slow-phase velocity peak) across patients in the acute stage (mean = 35%, range 25–45%) and return to normal values 6 months later (mean = 13%, range 5–20%). Patients who experienced benign paroxysmal positional vertigo as a trigger for PPPD had no symptoms or signs of active positional vertigo at the time of entry into the study. The duration of illness for patients with PPPD ranged from 8 to 120 months with a median of 18 months (see also Table 1). The severity of impairment due to dizziness was measured in patients with PPPD using the Dizziness Handicap Inventory (DHI) (Jacobson and Newman 1990). DHI scores ranged from 10 to 60, indicating a range of low to severe handicap with a mean ± SD of 34 ± 16.1. Generalized Anxiety Disorder (GAD-7) (Spitzer et al. 2006) scores in PPPD patients ranged from 1 to 18 with a mean ± SD of 8.86 ± 5.2. Only five patients had a score higher than the cut-off of 10. All patients were also evaluated with the Mini-International Neuropsychiatric Inventory (MINI) to detect active psychiatric illnesses. In a confirmatory analysis to exclude the effects of active psychiatric disorders, we removed five patients with PPPD who showed active psychiatric comorbidities when assessed with the MINI (Tables S1 in Supplementary Materials). Of note, none of the patients with psychiatric comorbidities were receiving psychoactive drugs.

**Table 1:** Demographic, clinical, and neuroimaging characteristics in patients with Persistent Postural Perceptual Dizziness (PPPD) and healthy controls

Demographic and clinical measures	PPPD patients (*n* = 15)	Healthy controls (n = 15)	Group differences
	Mean ± SD	Mean ± SD	χ^2^, T, *p*-values
Sex (Number of men & women)	9/6	7/8	χ^2^ = 0.53; *p* < 0.46
Age (years)	33.4 ± 12.4	30.1 ± 5.6	*T* = −0.92; *p* < 0.36
Generalized anxiety disorder scale (GAD-7)	8.8 ± 4.8	7.47 ± 4.5	*T* = −0.82; *p* < 0.42
Patient health questionnaire (PHQ-9)	8.6 ± 5.2	5.6 ± 5.0	*T* = −1.59; *p* < 0.12
NEO personality inventory – revised (NEO-PI-R) factors			
Neuroticism	56.2 ± 10.7	55.0 ± 9.8	*T* = −0.30; *p* < 0.76
Extraversion	51.1 ± 7.9	53.3 ± 10.2	*T* = 0.66; *p* < 0.51
Openness	45.2 ± 10.4	53.0 ± 10.1	*T* = 2.04; *p* < 0.05
Agreeableness	43.4 ± 8.4	47.5 ± 8.4	*T* = 1.31; *p* < 0.20
Conscientiousness	49.7 ± 8.7	49.6 ± 9.2	*T* = −0.03; *p* < 0.97
Total gray-matter volume (ml)	614.3 ± 62.4	631.8 ± 69.2	*T* = 0.73; *p* < 0.47
Total intracranial volume (ml)	1519.5 ± 185.6	1533.6 ± 227.1	*T* = 0.19; *p* < 0.85
Dizziness handicap inventory (DHI)	34.0 ± 17.1	N/A	–
Disease duration (months)	32.53 ± 37.2	N/A	

We enrolled fifteen healthy volunteers who were matched to the PPPD group in terms of sex, age, and scores on self-reports of generalized anxiety (GAD-7) and depression (Patient Health Questionnaire [PHQ-9]) (Kroenke et al. 2001). We selected healthy controls with overall personality profiles that matched our patients with PPPD based on a computerized version of the Italian translation of the revised version of the NEO personality inventory (NEO-PI-R) (Costa and McCrae 1997). All participants gave written informed consent to participate in this study, which was approved by a local ethical committee, in accordance with the declaration of Helsinki (http://www.wma.net/en/30publications/10policies/b3/).

### MRI scanning, MRI data quality control and processing

MRI brain scans were obtained from all participants using a 3 Tesla Unit with an 8-channel head coil (Discovery MR-750, General Electric, Milwaukee, WI). Head movements were minimized using foam pads around participants’ heads. The MRI protocol included a whole-brain T1-weighted scan [SPGR; Echo Time (ET) 3.7 ms, Repetition Time (TR) 9.2 ms, flip angle 12°, voxel size 1.0 × 1.0 × 1.0 mm^3^].

Images were first screened for scanner artifacts, motion abnormalities, and gross neuroanatomical alterations by a consultant neurologist and a consultant neuroradiologist. Next, the T1-weighted images were analyzed using Freesurfer software (version 5.3.0) (http://www.nmr.mgh.harvard.edu/martinos) to create anatomical surface models for statistical analyses (Dale et al. 1999; Fischl et al. 1999; Fischl and Dale 2000). For each participant, the processing pipeline included removal of non-brain tissue, transformation to Talairach space, segmentation of gray and white matter tissues, intensity normalization, tessellation of the gray/white matter boundaries, automated topology correction, and surface deformation. To map each participant to a common space, the surface representing the gray matter–white matter boundary was registered to an average cortical surface atlas by using a non-linear procedure that optimally aligned sulcal and gyral features across participants.

Cortical thickness was defined by the shortest distance between the gray/white matter border and pial surfaces. Vertex-based estimates of SA were obtained by computing the average of the area of the triangles incident to that vertex (Dale et al. 1999; Fischl et al. 1999; Fischl and Dale 2000). Cortical volume (CV) was defined as the product of CT and SA. To calculate the LGI, an additional outer hull layer that tightly wrapped the pial surface was defined. Next, the LGI value at each vertex was computed within 25-mm circular regions of interest and represented by the ratio of the pial to outer hull surfaces (Schaer et al. 2012). All images were inspected visually to check for reconstruction errors including skull-strip errors, gross segmentation problems, and inaccuracies in the white-matter and pial surface reconstruction. Surface inaccuracies were corrected manually with Freesurfer’s editing tools. Edited images were re-processed through the Freesurfer pipeline. This cycle was repeated until all surface errors were corrected.

### Statistical analyses

A general linear model (GLM) was used to identify between-group differences in CT, SA, cortical volume, and LGI. For the PPPD group, correlations between subject specific SBM measures at each vertex and individual DHI scores and illness duration were also assessed for each hemisphere. To control for multiple comparisons, cluster correction was completed using Monte Carlo simulation (vertex-wise cluster forming threshold of *p* < 0.05) at a cluster-wise *p*-value (CWP) of p < 0.05 (Hagler et al. 2006). Age and gender were included as covariates of no interest in all analyses. Individual mean CT values were used as nuisance variables in the CT analysis while total intracranial volume was considered as a variable of no interest in analyses that involved SA and cortical volume as outcome measures. Total SA was chosen as a covariate in the LGI analysis as there is an evidence that it has linear relationship with gyrification (Luders et al. 2006). In CT, SA, and CV analyses, a smoothing kernel of 5-mm Full Width at Half Maximum (FWHM) was used. In the gyrification analyses, no smoothing kernel (FWHM = 0 mm) was employed because the LGI implemented in Freesurfer is already relatively smoothed by default (Schaer et al. 2012).

## Results

### Demographics, clinical variables, and general structural neuroimaging data

Table 1 summarizes the demographics, clinical variables, and general structural neuroimaging data (e.g. total intracranial volume) for the PPPD and healthy control groups. There were no significant differences in mean age, sex distribution, or mean scores on the GAD-7, PHQ-9, or in four of the five NEO-PI-R personality factors. Although scores for openness differed statistically between groups, both patients and controls had mean scores within the normative range for the general population (standardized scores of 45–55). This implies that the NEO-PI-R profiles of both groups was reasonably matched for personality traits. Likewise, there were no significant differences in total gray matter volume or intracranial volume between patients with PPPD and healthy controls indicating a good match between subject groups on these demographic, psychological, and general anatomical variables.

### Relationship between cortical morphology and dizziness severity or disease duration

**Fig. 1:**
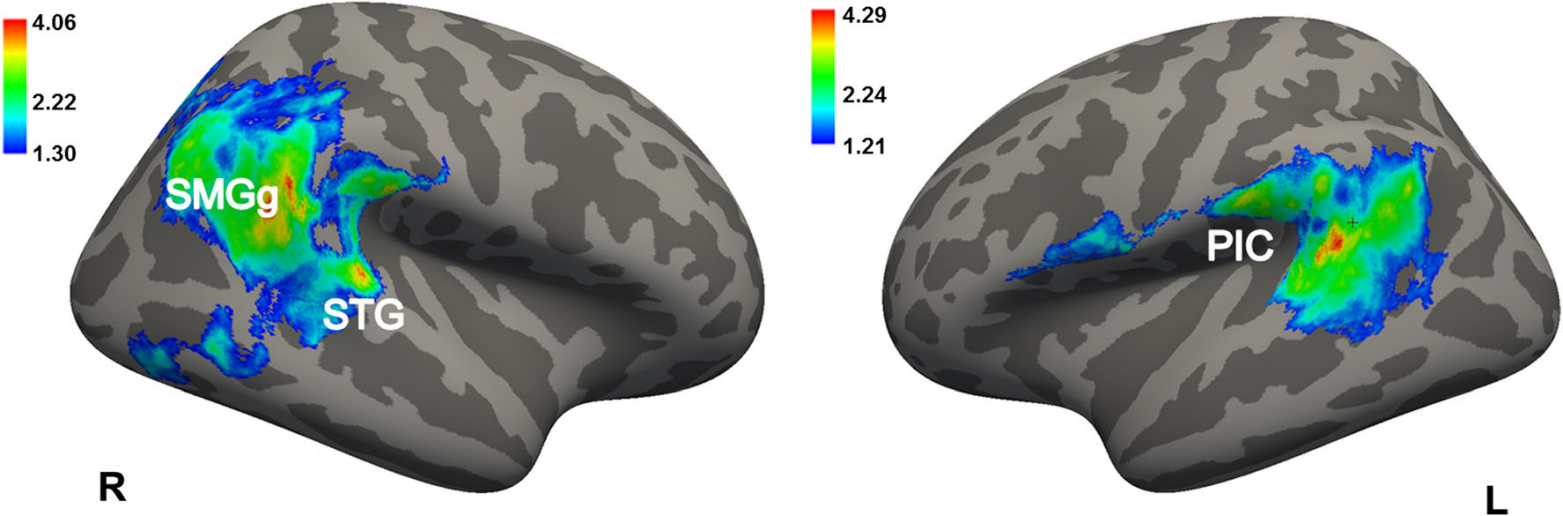
Cortical areas showing significantly decreased local gyrification index in PPPD patients relative to healthy controls. Labels refer to peaks: SMGg, Supra-marginal gyrus; STG, Superior Temporal Gyrus (posterior part) and PIC, Posterior Insular Cortex. Color bar represents -log_10_ (*P* value). R, L, right/left hemisphere

**Fig. 2:**
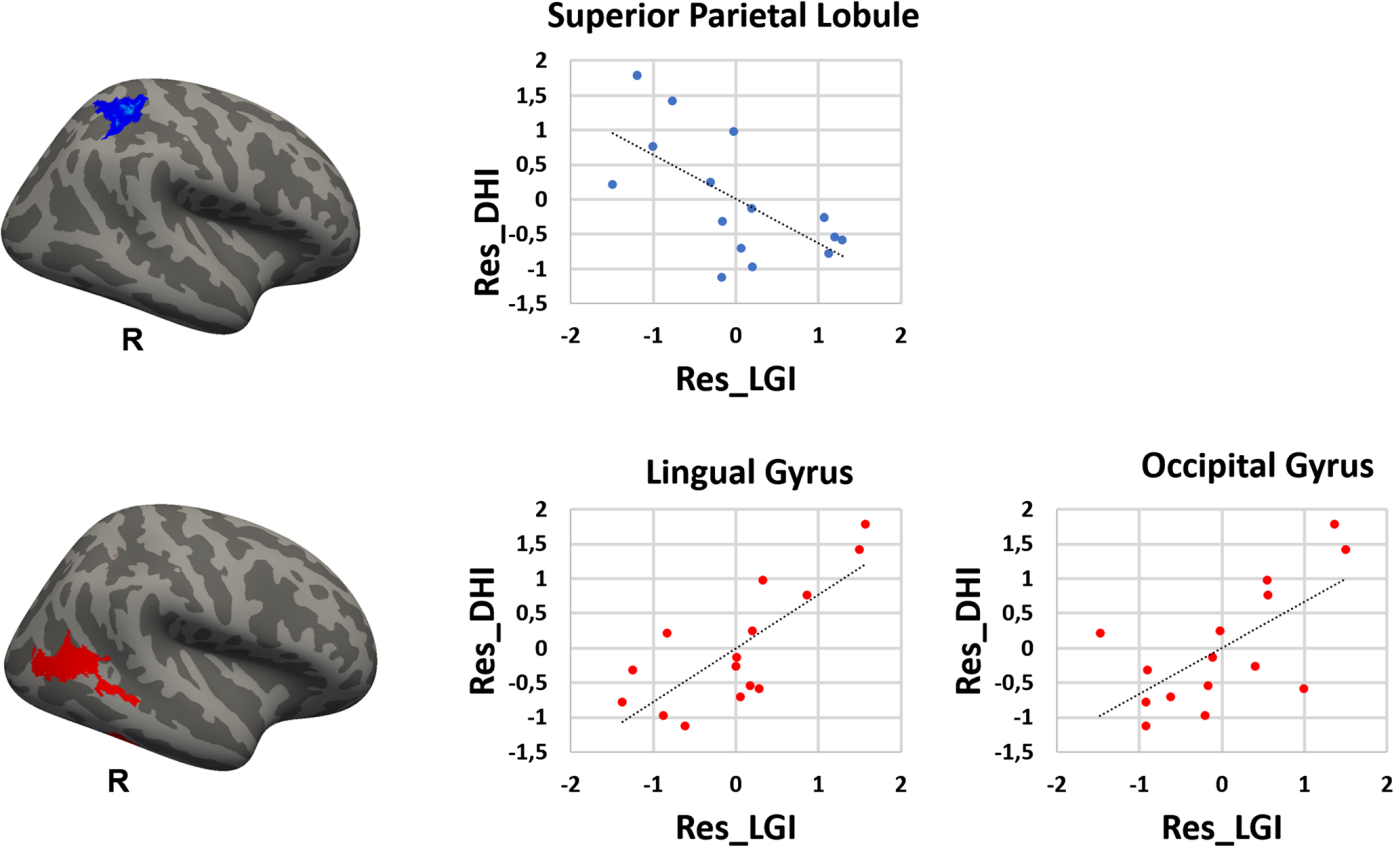
Cortical areas showing significantly negative (in blue) and positive (in red) correlation between Dizziness Handicap Inventory (DHI) and local gyrification index in Persistent Postural Perceptual Dizziness (PPPD) patients. Residual (Res_) scores are shown in the Y and X axes

**Fig. 3:**
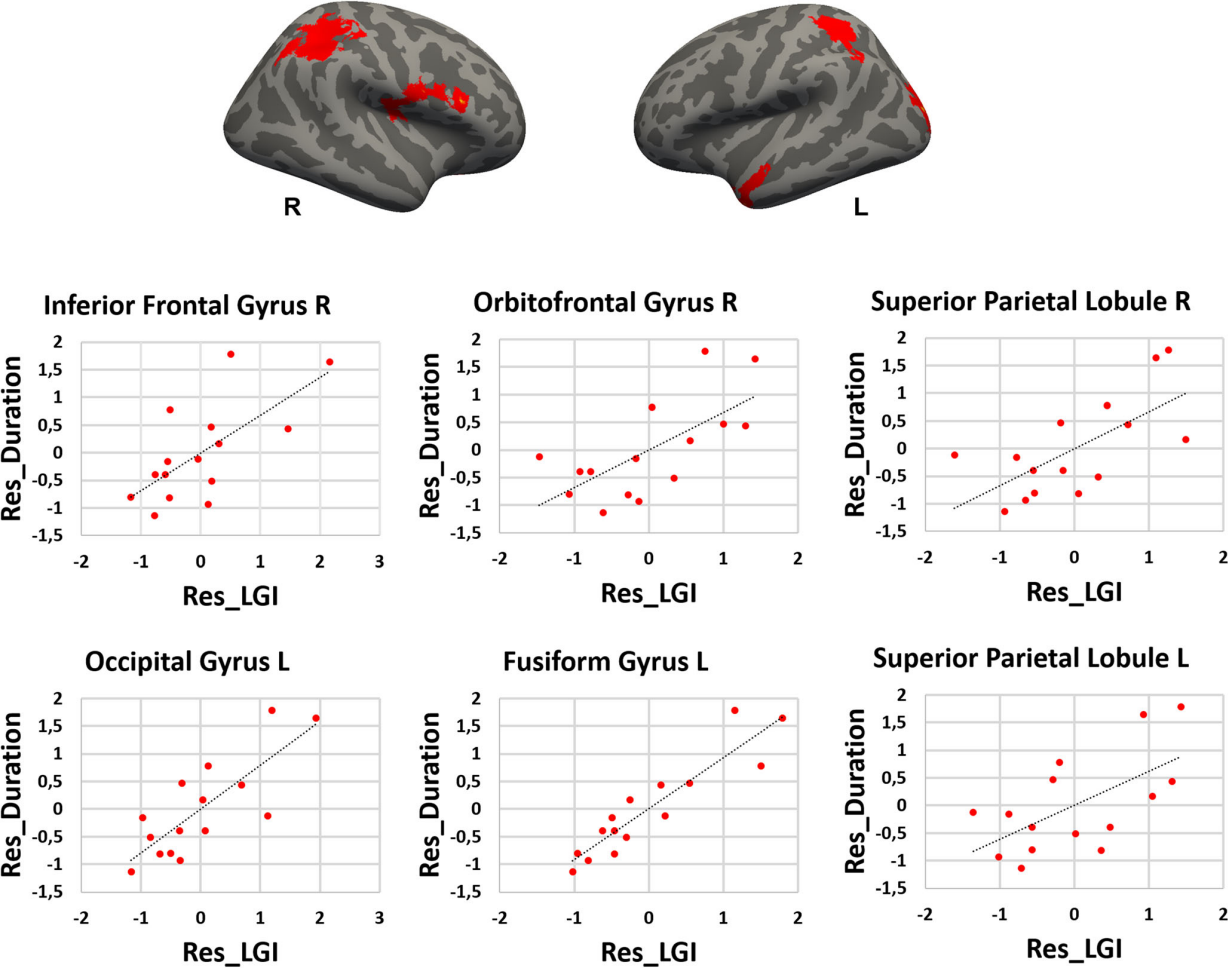
Cortical areas showing significantly negative (in blue) and positive (in red) correlation between illness duration and local gyrification index in Persistent Postural Perceptual Dizziness (PPPD) patients. Residual (Res_) scores are shown in the Y and X axes

Relative to healthy controls, patients with PPPD had significantly decreased mean values for the LGI in the posterior insular cortex, superior temporal gyrus, superior temporal sulcus, supra-marginal gyrus, precentral gyrus bilaterally, as well as in the inferior and superior parietal gyri, pre-cuneus, cuneus, inferior and middle temporal gyri and lateral occipital gyrus in the right hemisphere, and finally in the post-central gyrus and parietal operculum in the left hemisphere (Fig. 1, Table 2). Most of these results were confirmed when removing the PPPD patients with psychiatric comorbidities from the main analyses. In particular, PPPD patients without psychiatric comorbidities continued to show decreased LGI in the superior and middle temporal pole gyri, supra-marginal gyrus and lateral occipital gyrus in the right hemisphere (see Table S1, Supplementary Materials). There were no significant differences between groups in CT, SA, or cortical volumes. In the PPPD group, DHI scores correlated positively with the LGI in the right lingual gyrus and right lateral occipital gyrus and negatively with the LGI in the right superior parietal lobule (Fig. 2, Table 3). Disease duration correlated positively with the LGI in the right lateral orbitofrontal gyrus, right superior parietal gyrus, right inferior frontal gyrus (pars opercularis), left lateral occipital gyrus, left fusiform gyrus, and left superior parietal gyrus (Fig. 3).

**Table 2:** Cortical areas displaying significantly decreased local gyrification index in patients with Persistent Postural Perceptual Dizziness (PPPD) relative to healthy controls (HCs). Whole-brain local gyrification index results derived from FreeSurfer. Correction for multiple comparisons was performed using Monte Carlo simulation (vertex-wise cluster forming threshold of p < 0.05) at a cluster-wise p-value (CWP) of *p* < 0.05. Age and gender were included as covariates of no interest. CWP, cluster-wise P corrected level

Local gyrification index				
HCs > PPPD				
Hemisphere	Max	Size	CWP	Regions
Left	4.29	3461.1	<0.001	Superior Temporal Gyrus
				Superior Temporal Sulcus Posterior insula
				Post-central Gyrus
				Supra-marginal Gyrus
	2.44	545.7	0.025	Pre-central Gyrus
				Post-central Gyrus
				Inferior Frontal Gyrus (pars opercularis)
Hemisphere	Max	Size	CWP	Regions
Right	4.06	8919.1	<0.001	Supra-marginal Gyrus
				Pre-central Gyrus
				Superior Temporal Gyrus
				Superior Temporal Sulcus Posterior insula
				Inferior Parietal Cortex
				Superior Parietal Cortex
				Pre-cuneus
				Cuneus
				Inferior Temporal Gyrus
	2.42	696.8	<0.001	Middle Temporal Gyrus
				Lateral Occipital Gyrus

**Table 3:** Cortical areas showing significantly positive and negative correlation between local gyrification index (LGI) and clinical variables (Dizziness Handicap Inventory (DHI) and illness duration) in Persistent Postural Perceptual Dizziness (PPPD) patients. Whole-brain local gyrification index results derived from FreeSurfer. Correction for multiple comparisons was performed using Monte Carlo simulation (vertex-wise cluster forming threshold of p < 0.05) at a cluster-wise p-value (CWP) of p < 0.05. Age and gender were included as covariates of no interest. CWP, cluster-wise P corrected level

LGI – DHI				
Positive correlation				
Hemisphere	Max	Size (mm^2^)	CWP	Regions
Right	2.72	966.3	0.002	Lateral Occipital Gyrus
	2.33	1978.7	<0.001	Lingual Gyrus
Negative correlation				
Hemisphere	Max	Size (mm^2^)	CWP	Regions
Right	−2.22	874.6	0.004	Superior parietal Lobule
LGI – Illness duration				
Positive correlation				
Hemisphere	Max	Size (mm^2^)	CWP	Regions
Right	3.697	1073.9	<0.001	Inferior frontal Gyrus, pars opercularis
	3.010	1109.2	<0.001	Lateral Orbitofrontal Gyrus
	2.401	2734.0	<0.001	Superior parietal Lobule
Left	3.848	1519.6	<0.001	Lateral Occipital Gyrus
	3.589	2608.4	<0.001	Fusiform Gyrus
	2.321	1064.2	0.002	Superior parietal Lobule

## Discussion

In this study, we used SBM methods to measure cortical morphology in patients with PPPD and to examine relationships among cortical thickness, surface area, local gyrification index (cortical folding), and severity and duration of dizziness. We found that patients with PPPD, relative to healthy controls, had decreased cortical folding in key brain regions that comprise the posterior insula, superior temporal gyrus and sulcus, and supra-marginal gyrus. Decreased cortical folding extended to parietal and temporo-occipital association areas, specifically the inferior and superior parietal gyrus, pre-cuneus, cuneus, inferior and middle temporal gyri, and lateral occipital gyrus, in the right hemisphere, which is the dominant hemisphere for vestibular function in right-handed individuals. The regions surrounding the posterior Sylvian fissure and extending into adjacent temporal, parietal and occipital association areas have been strongly implicated in processing and integrating multi-sensory inputs from vestibular, visual, and somatosensory systems (Brandt 1999; Guldin and Grüsser 1998; Indovina et al. 2005; Lacquaniti et al. 2013; Lopez et al. 2012; Lopez and Blanke 2011; zu Eulenburg et al. 2012). They also play important roles in processing data related to motion of self, body posture, location, and movements of external objects (Panizzon et al. 2009; Schuez and Miller 2003; White et al. 2010), allowing highly mobile animals like humans to construct coherent, internal maps of spatial orientation and motion of self and objects in the environment (Indovina et al. 2013, 2016; Lacquaniti et al. 2013; Riccelli et al. 2017a, b; Schuez and Miller 2003; White et al. 2010).

We observed no effects for CT and SA which suggests that cortical folding alterations may be the primary structural marker of PPPD. There may also be an important relationship between areas with decreased LGI that we identified in this study and regions with reduced functional connectivity that we found in our previous fMRI study of this same patient cohort. Structural and functional abnormalities corresponded in the right vestibular cortex, specifically in the right superior temporal gyrus. The significance of these parallel results lies in the tension-based theory of morphogenesis, which posits that folding of the cortical mantle is a consequence of mechanical tension along the axons that connect different brain regions (Van Essen 1997). Hence, reduced cortical folding may result from weakening of network connectivity across posterior temporo-parietal cortical areas centered around the superior temporal gyrus. Our findings of both reduced gyrification and altered connectivity in this key component of the dominant vestibular cortex amplifies our previous suggestion that alterations in activity and connectivity in this region may underlie the core symptoms of persistent unsteadiness and dizziness as well as their exacerbation by upright posture in patients with PPPD (Staab 2012). The folding alterations observed in the superior temporal gyrus, middle temporal gyrus and right precentral gyrus also confirm the gray matter alterations reported in these regions in a recent VBM study (Wurthmann et al. 2017) .

It is not possible to determine from a cross-sectional study whether the changes in cortical folding are a primary or secondary phenomena. In the case they predate the onset of PPPD, they may represent a structural risk factor, that is, an area of vulnerability in the brain that limits healthy recovery following exposure to factors that precipitate PPPD. Alternatively, the SBM changes in PPPD that we identified in this study may develop as a result of changes in physiological functioning that are hypothesized to be key mechanisms of PPPD, that is, areas of brain plasticity resulting from altered postural control and changes in multi-sensory space-motion information processing.

The significant correlations between LGI and clinical features such as the DHI scores and disease duration in patients with PPPD offer further clues regarding the additional neural mechanisms that may contribute to the core symptoms of the disorder and their exacerbation by moving or complex visual stimuli. DHI scores positively related with the cortical folding in the right lingual gyrus and the lateral occipital gyrus, while they were negatively related to cortical folding in the right superior parietal lobule. These results are consistent with physiologic studies of patients with persistent visually induced dizziness triggered by various structural vestibular disorders (Bronstein 2005, 2004; Bronstein et al. 2013; Cousins et al. 2014; Guerraz et al. 2001) and with computerized dynamic posturographic measurements in patients with PPPD (Ödman and Maire 2008; Söhsten et al. 2016), which demonstrated over reliance on visual stimuli for perception of verticality and control of posture (i.e., visual dependence). Thus, structural and functional alterations in the multimodal vestibular cortex coupled with structural changes in opposite directions in visual and somatosensory association areas which are important for processing spatial information may underlie the phenomenon of visual dependence and its clinical manifestation as hypersensitivity to complex or moving visual stimuli in patients with PPPD.

Curiously, we found no differences in cortical morphology between PPPD and healthy controls in anterior regions of the brain that are involved in modulating anxiety or threat-related behaviors. This can be due to the fact that our PPPD and control groups were closely matched on psychological variables so that our between group analyses would not have detected abnormalities in cortical structure if they were related solely to these psychological variables.

Some limitations of this study should also be acknowledged. First, SBM metrics do not allow characterization of gray matter abnormalities that might be present at the subcortical level. Second, we examined a relatively small number of patients with PPPD which implies that replication in larger samples is warranted. Third, our groups were not perfectly matched on age and sex although there were no statically significant differences in these demographic variables. Fourth, this is a cross-sectional study which means that future research will have to examine patients with PPPD prospectively to ascertain when the brain morphological changes identified here develop, i.e., if they are primary or secondary alterations to the initial event that triggers PPPD. It will also be important to assess in longitudinal studies the extent to which the brain structural or function alterations in PPPD can be modified by available treatments for PPPD. Last but not least, forthcoming studies should include groups of patients with psychiatric and neuro-otological disorders who have not developed PPPD to confirm and extend the current findings and demonstrate their specificity to this disorder.

## Conclusions

In this study, we used surface based morphometry to assess the structural integrity of the cortical mantle in 15 patients with PPPD, a chronic functional vestibular disorder. The results of this structural neuroimaging study extended the findings of our previous functional neuroimaging investigation of the same cohort and structural and functional imaging studies reported by other investigators. Here we showed that patients with PPPD, compared to 15 well-matched healthy controls, had abnormal cortical folding in regions of the brain that comprise the multi-modal vestibular cortex bilaterally and also in adjacent temporo-parietal areas that are involved in processing space and motion information in the right hemisphere. In the PPPD group, we also found significant associations between severity of dizziness handicap and increased gyrification in two visuo-spatial areas. Furthermore, we found decreased gyrification in a somatosensory-spatial area of the parietal cortex, which is consistent with the hypothesis that increased visual dependence is an important pathophysiologic process in PPPD. Despite extensive clinical data showing that anxiety-related personality traits are risk factors for PPPD and that high anxiety during acute vestibular symptoms plays an important role in its development, we did not find any structural abnormalities in cortical regions that modulate anxiety and threat responses.

## Electronic supplementary material

Supplementary Table S1 (DOCX 14 kb)
