# Supplementary material for: Reduced cortical folding in multi-modal vestibular regions in persistent postural perceptual dizziness
Source: Brain Imaging Behav. 2018 Jun 2;13(3):798–809. doi: 10.1007/s11682-018-9900-6 (PMC6538588; doi:10.1007/s11682-018-9900-6)
Supplement: Supplementary file 1 — (DOCX 14 kb) [file 11682_2018_9900_MOESM1_ESM.docx]

**Table S1.** Cortical areas displaying significantly decreased local gyrification index in patients with Persistent Postural Perceptual Dizziness (PPPD) (excluding patients with psychiatric comorbidity) relative to healthy controls (HCs). Whole-brain local gyrification index results derived from FreeSurfer. Correction for multiple comparisons was performed using Monte Carlo simulation (vertex-wise cluster forming threshold of p<0.05) at a cluster-wise p-value (CWP) of p<0.05. Age and gender were included as covariates of no interest. CWP, cluster-wise P corrected level.

| **LOCAL GYRIFICATION INDEX** | | | | | |
| --- | --- | --- | --- | --- | --- |
| **HCs > PPPD** | | | | |  |
| ***Hemisphere*** | ***Max*** | ***Size*** | ***CWP*** | ***Regions*** |  |
| Left | 3.11 | 575.8 | <0.017 | Posterior cingulate gyrus  Isthmus of cingulate gyrus |  |
|  | 2.58 | 1036.7 | <0.001 | Lingual gyrus |  |
|  |  |  |  | Fusiform gyrus |  |
| ***Hemisphere*** | ***Max*** | ***Size*** | ***CWP*** | ***Regions*** |  |
| Right | 3.11 | 558.4 | <0.008 | Supra-marginal Gyrus |  |
|  | 2.58 | 867.6 | <0.001 | Lateral Occipital Gyrus |  |
|  | 2.54 | 554.1 | 0.007 | Superior Temporal Gyrus  Middle Temporal Gyrus |  |
|  |  |  |  |  |  |
| **PPPD > HCs** | | | | |  |
| ***Hemisphere*** | ***Max*** | ***Size*** | ***CWP*** | ***Regions*** |  |
| Left | 2.87 | 2820.4 | <0.001 | Superior Temporal Gyrus |  |
|  |  |  |  | Temporal Pole |  |
|  |  |  |  |  |  |
